# Effects of pre-sowing seed treatments on establishment of dry direct-seeded early rice under chilling stress

**DOI:** 10.1093/aobpla/plw074

**Published:** 2016-11-07

**Authors:** Weiqin Wang, Shaobing Peng, Qian Chen, Junhao Mei, Huanglin Dong, Lixiao Nie

**Affiliations:** 1 National Key Laboratory of Crop Genetic Improvement, MOA Key Laboratory of Crop Ecophysiology and Farming System in the Middle Reaches of the Yangtze River, College of Plant Science and Technology, Huazhong Agricultural University, Wuhan, Hubei 430070, China; 2 Hubei Collaborative Innovation Center for Grain Industry, Yangtze University, Jingzhou, Hubei 434023, China

**Keywords:** Chilling stress, dry direct-seeded early rice, seed coating, seed priming, starch metabolism

## Abstract

Mechanization and simplification are inevitable trends in agriculture production to decrease input demands and simultaneously improve resource use efficiency. Dry direct-seeded rice is a resource-saving cropping system and has been considered as a replacement for traditional transplanted rice. However, the poor establishment of dry direct-seeded early rice, primarily induced by chilling stress, has limited the wide adoption of this system. To examine the effects of pre-sowing seed treatments on rice germination, seedling growth and associated metabolic events under chilling stress, two seed coating treatments (Hanyubaomu coating and Miaoboshi coating) and two seed priming treatments (selenium priming and salicylic acid priming) were tested in field and growth chamber experiments. The results revealed that under chilling stress, seed priming increased the rice seed germination by 20.96–26.31 %. The length and weight of shoots and roots were also significantly increased. The two seed coating treatments were not effective in enhancing seed germination and seedling growth under chilling stress. The improved germination and seedling growth of primed seeds under chilling stress were strongly linked with higher α-amylase activity and total soluble sugar content. Conclusively, these findings will provide new avenues for understanding and advancing pre-sowing seed treatments of dry direct-seeded early rice.

## Abstract (precis)

Our study revealed that priming treatments significantly enhanced the seed germination and seedling growth of dry direct-seeded early rice under chilling stress. The improved emergence and vigorous seedling growth induced by the seed priming treatments were associated with increased α-amylase activity and total soluble sugar contents in the primed seedlings. These findings will have practical implications for the sustainability and productivity of dry direct-seeded early rice.

## Introduction

Global agriculture production systems are threatened by climate change, resource shortages and pressure from the ever-increasing food demand. It has been estimated that by 2050, the world population is projected to increase to 9.5 billion and the grain production must increase by 1 % annually to meet the growing demand for food (Rosegrant *et al.* 1995). Rice (*Oryza sativa*) is the prominent cereal foodstuff and staple food for more than half of the world’s population, especially in tropical Latin America and East, South and Southeast Asia (Seck *et al.* 2012). In the subtropical climates, rice can be grown up to 2 times per year on the same field. The wide adoption of double-season rice systems in Asia increases multiple cropping index expressed as a ratio between total sown area a year and the area of the cultivated land and thus contributes substantially to global rice supply (Ray and Foley, 2013). Transplanting is the major rice establishment method and 77 % of rice is transplanted globally (Rao *et al.* 2007). But the conventional transplanted rice system demands a large amount of water, energy input and labour. Water scarcity governed by climate change is becoming a major threat for sustainability and productivity of this production system. Thus, the traditionally transplanted rice is no longer suitable for sustainable development, suggesting that it is inevitable to shift the traditional rice production method to mechanized and intensive rice production system. Dry direct-seeded rice has emerged as an alternative option for rice production and refers to the process of establishing the crop from seeds sown in nonpuddled and unsaturated soil rather than transplanting seedlings in a puddled field (Liu *et al.* 2015). Compared with conventional transplanted rice, dry direct-seeded rice has the advantages of saving water and labor, and it is more suitable for the development of mechanization (Kumar and Ladha, 2011).

Incorporating dry direct seeding into double season rice systems to develop ‘double dry direct-seeded rice’ (refers to the process of crop establishment by dry-direct-sowing the seeds in the field in both early and late seasons)could be a promising strategy to greatly increase resource use efficiency, the multiple-crop index and total grain production (Yang *et al.* 2010). Nevertheless, the poor crop establishment of dry direct-seeded early rice induced by chilling stress is one of the major constraints to the large-scale adoption of double dry direct-seeded rice. In subtropical regions, double dry direct-seeded rice cropping is usually practiced with an early-season crop from April to July and a late-season crop from July to October. To ensure safe production, early rice must be directly sown in early or mid-April, when the temperature is generally low. It has been reported that the daily mean temperature in April is 16.4 °C, and cold spells (temperature <10 °C lasting for >3 days) occur frequently in central China (Ma *et al.* 2011).

Chilling stress significantly reduced seed germination, seedling emergence and the subsequent growth of rice seedlings (Yoshida, 1981; Sipaseuth *et al.* 2007; Guan *et al.* 2009; Ye *et al.* 2009). It has been reported that chilling stress reduced the seedling emergence of direct-seeded early rice by 38–55 % in central China (Zhang, 2012). The poor germination of rice seeds under chilling stress was associated with damage to the plasma membrane, which would result in the leakage of solutes such as amino acids and carbohydrates from the rice seeds (Cruz and Milach, 2004). The low temperature depressed the water uptake of the root, interrupted the cellular elongation and division and leaded to metabolic imbalance of the rice seedlings, thus reduced the growth of the shoots and roots (Lyons, 1973; Zhao *et al.* 2013). A technology that could enhance the seed germination and seedling growth of early direct-seeded rice under chilling stress is desperately needed.

Several studies have reported that ‘pre-sowing seed treatments’ such as seed priming and seed coating could improve the abiotic stress resistance of plants. Seed priming is a technique by which seeds are partially hydrated to a point where germination-related metabolic processes begin, but radicle emergence does not occur (Heydecker, 1977; Bradford, 1986). It is among the most effective and practical short-term approaches for increasing seed vigour and the synchronization of germination under different stresses (Kaya *et al.* 2006). It has been reported that seed priming significantly improved rice seed germination and early seedling growth under abiotic stresses, such as drought (Lee *et al.* 1998; Zheng *et al.* 2016), salinity (Ruan *et al.* 2003), waterlogging (Sasaki *et al.* 2005) and low temperature (Zhang *et al.* 2006). Seed coating is a technique in which various materials, such as fertilizers, nutritional elements, moisture attractive or repulsive agents, plant growth regulators, rhizobium inoculum, chemicals and pesticides are added to the seed surface by adhesive agents (Scott, 1989). Several studies have reported that seed coating could enhance germination, promote root and shoot growth and increase stress resistance (Hirano *et al.* 2001; Sato and Maruyama, 2002; Cheng, 2008). Positive effects of seed coating on seed germination and seedling growth under chilling and drought stresses have also been documented (Zhang *et al.* 2011; Zhu and Wang, 2013). However, the effects of these pre-sowing seed treatments on seed germination and seedling growth under chilling stress in dry direct-seeded early rice remain unknown.

Starch metabolism has been considered to play a key role in early seedling vigour. Williams and Peterson (1973) have reported that the α-amylase activity and total soluble sugar contents in rice seeds were strongly linked to the seed germination and seedling growth of rice. The results of Lee and Kim (2000) showed that seed priming was helpful in enhancing starch metabolism in rice seeds. Therefore, the activity of starch metabolism should be an important trait reflecting seedling vigour, particularly under stress conditions.

This study examined the effects of seed coating and priming treatments on dry direct-seeded early rice under both field and growth chamber conditions and explore starch metabolism changes during seed germination induced by pre-sowing seed treatments in response to chilling stress. Finally, the optimal ‘pre-sowing seed treatments’ can be identified to secure good crop establishment in dry direct-seeded early rice systems in central China.

## Methods

### Seed source

Seeds of two widely grown indica rice (*Oryza sativa*) cultivars, Huanghuazhan (HHZ, inbred) and Yangliangyou-6 (YLY6, hybrid), were obtained from the Crop Physiology and Production Center, Huazhong Agricultural University, Wuhan, China. Both cultivars have initial germination of > 95 % at 25 °C. The initial seed moisture content was <10.0 % (on a dry weight basis). HHZ was used in the field experiment, and both cultivars were used in the growth chamber experiments. All seeds used in this study were selected from the same lot.

### Seed coating treatment

Seed coating treatment is the process of directly wrapping the coating agents on the dry rice seeds without seed soaking. Two commercial rice coating agents, Hanyubaomu (HYBM, produced by Jiangsu Lixiahe Agriculture Research Institute) and Miaoboshi (MBS, produced by Heartale Chemurgy Co. Ltd, Hunan Agriculture University), were selected as seed coating treatments for this study. The two seed coating treatments were widely used by rice farmers in direct seeding and the effectiveness of HYBM and MBS in enhancing crop establishment was documented (Chen *et al.* 2005; Xiao, 2010; He *et al.* 2011). But their effects on rice seeds germination and seedling establishment under chilling stress have not been studied. The HYBM coating agents used in this study were composed of carbendazim, paclobutrazol and mineral clay, and the components of the MBS coating agents were prochloraz, imidacloprid, paclobutrazol and polymer materials. The ratio of seed weight to coating agent weight was 8:1 for HYBM (He *et al.* 2011) and 40:1 for MBS (recommended by the producer). For the HYBM-coating treatment, the seeds were soaked in distilled water for 20 min, and then the excess water was blotted up using blotting paper. The coating agents and seeds were then placed in a round-bottomed container, where the seeds and the coating agents were stirred until the agents were evenly distributed on the seeds. For the MBS-coating treatment, the coating agent was mixed with distilled water at a weight to volume (w/v) ratio of 1:1. Dry seeds and the coating agent/water mixture were mixed in a round-bottomed container until the agents were evenly distributed on the seeds. Then, the coated seeds were transferred to an air drying oven at 25 °C for 24 h to reduce the moisture contents. After drying, the seeds were sealed in plastic bags and stored in a freezer (−4 °C).

### Seed priming treatment

Different from the seed coating treatment, the process of seed priming is soaking the dry seeds into the solution which contained priming agent for 24 h and then air-dried until the seed moisture content reduced to <10 %. Two seed priming treatments were selected via preliminary experiments. Previously, we compared the effectiveness of different seed priming techniques including hydro-priming (primed with water), osmo-priming (CaCl_2_, calcium chloride), redox priming (H_2_O_2_, hydrogen peroxide), nutri-priming (Se, selenium) and hormonal-priming (SA, salicylic acid) with different priming reagent concentrations under chilling stress conditions and found that Se and SA priming were the most effective among these priming treatments. Therefore, the seed priming treatments selected for this study were Se (50 µM sodium selenite) and SA (100 mg L ^−^ ^1^ salicylic acid). Seeds were primed in the dark at 25 °C for 24 h, with constant gentle agitation. The ratio of seed weight to solution volume (w/v) was 1:5, and the priming solution was changed after every 12 h. After 24 h, the primed seeds were washed with distilled water for 2 min, surface dried and transferred to an air drying oven at 25 °C for 48 h to reduce the moisture contents to <10 %. After drying, the seeds were sealed in plastic bags and then stored in a freezer (−4 °C).

### Experimental design

***Field experiment.*** The field experiment was performed at the experimental station of Zhougan Village, Dajin Town, Wuxue Country, Hubei Province, China (29°51′N 115°33′E). Soil samples from the upper 20 cm layer were collected for analysis of soil chemical properties. The soil pH, organic C, available P, available K and total N content were 5.29, 23.35 g kg ^−^ ^1^, 7.85 mg kg ^−^ ^1^, 84.17 mg kg ^−^ ^1^ and 0.17 %, respectively. The experiment was arranged in a randomized complete block design with four replications. The treatments were no-priming and no-coating control (CK), Hanyubaomu coating (HYBM coating), Miaoboshi coating (MBS coating), selenium priming (Se priming) and salicylic acid priming (SA priming). The inbred rice variety HHZ was used in the field experiment. Before sowing, the soil was dry ploughed and harrowed without puddling. Coated and primed rice seeds were manually sown at a 20-cm row to row and 5-cm plant to plant distance on 13th April 2015. The sowing depth was ∼2–5 cm, and the seeds were covered with soil immediately after sowing. During seed germination, plots were flash irrigated with ∼5-cm water each time when the soil moisture tension at 15 cm depth reached −15 kPa. The soil temperature was constantly recorded one day after sowing (1 DAS) using a HOBO data logger with three detectors. The detectors were buried at a soil depth of 5 cm.

The emergence of seeds was recorded on a daily basis according to Association of Official Seed Analysis (1990) and expressed as a percentage. At 12 DAS, 10 seedlings from each plot were carefully sampled. After measuring shoot length and maximum root length, all seedlings were dissected into root and shoot for the determination of root and shoot fresh weight.

***Growth chamber experiment.*** To examine the biochemical and physiological changes underlying the seed coating and seed priming treatments, a growth chamber experiment was conducted in the Crop Physiology and Production Center, Huazhong Agricultural University, Wuhan, China. Plastic trays 21.0 cm × 17.0 cm × 15.0 cm in size were filled with 2 kg of soil collected from the field where field experiment was conducted. The soil water content (dry weight basis) was kept at 20 %. The pre-sowing seed treatments were the same as the treatments in the field study. Two cultivars, HHZ and YLY6, were used in this study to verify that the intrinsic changes in the rice induced by the pre-sowing seed treatments were similar for different cultivars.

In each tray, 10 rows (two rows for each treatment) of rice seeds were evenly sown, 10 seeds for each row. After sowing, the seeds were immediately covered with soil. The sowing depth was kept the same with that in the field experiment. The experiment was laid out in a completely randomized design with a factorial arrangement replicated 8 times. Four replications of trays were used to record seed germination and seedling growth parameters. The seedlings in the other four replications were sampled for the determination of α-amylase activity and soluble sugar contents. All the trays were placed in a growth chamber with a 12-h light period, 19.6 °C day and 13.4 °C night temperatures according to the mean day-time and night-time temperatures recorded during 1–10 DAS in the field study. The soil water content in the plastic trays was not controlled rigorously, but frequent irrigation made sure that plants did not experience drought stress and no standing water was kept in the plastic trays throughout the experiment.

The germination of seeds was recorded on daily basis according to AOSA (1990) until a constant count was achieved. Seeds were considered to have germinated when the radicle and hypocotyl length exceeded 2 mm. Germination percentage was taken as the ratio of the number of seeds germinated to the total number of seeds sown and is expressed as a percentage. At 12 DAS, 10 seedlings were randomly sampled from each treatment and each replication to record their shoot and root length. These seedlings from each replicate were then dissected into roots and shoots, and their fresh weight was recorded immediately.

For the determination of α-amylase activity, 1.0 g dry seeds (0 DAS) and seedling samples at 3, 6 and 9 DAS, including shoot and root, were ground and mixed with 100 ml distilled water, allowed to stand for 24 h at 4 °C and then filtered with Whatman No. 42 filter paper. The enzyme activity was determined by the dinitrosalicyclic acid (DNS) method (Bernfeld, 1955). To determine the total soluble sugar contents, 0.5 g dry seeds (0 DAS) and seedling samples at 3, 6 and 9 DAS were ground and mixed with 50 ml distilled water, then left for 24 h at 4°C (Lee and Kim, 2000). This mixture was also filtered with Whatman No. 42 filter paper. The total soluble sugar contents were determined by the phenol sulphuric method (Dubois *et al.* 1956).

### Statistical analysis

All the data from the field and growth chamber experiments were analysed using Statistix 9.0. In the field experiment, we used one-way ANOVA, with pre-sowing seed treatments as grouping factor, germination, root length, shoot length, root fresh weight and shoot fresh weight as the response variable. The effect of pre-sowing seed treatments under growth chamber conditions was evaluated using two-way ANOVAs, with varieties and pre-sowing seed treatments as grouping factors, germination, root length, shoot length, root fresh weight, shoot fresh weight, α-amylase activity and total soluble sugars as response variables. The differences were analysed using the Least Significance Difference (LSD) test at the 0.05 probability level.

## Results

### Field experiments

**Figure 1:**
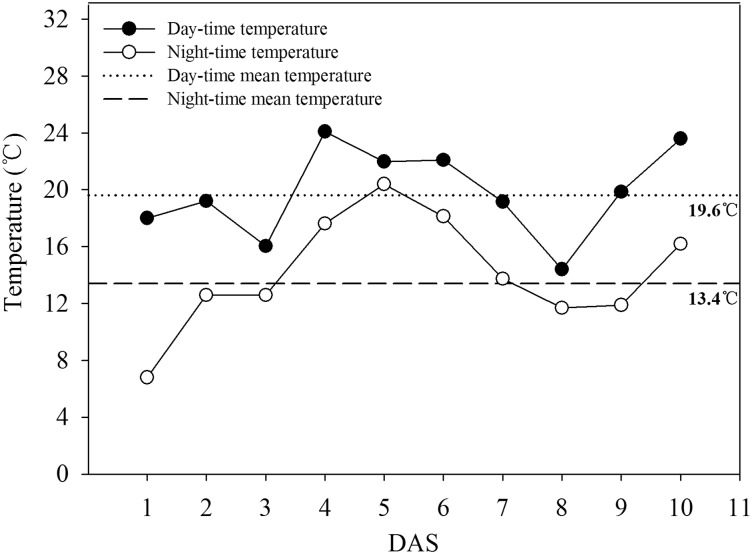
Day-time, night-time, day-time mean and night-time mean soil temperatures during seed germination (1–10 DAS) of dry direct-seeded early rice in field experiment. DAS, days after sowing. The upper straight line represents the day-time mean temperature (19.6 °C) during seed germination; the lower straight line represents the night-time mean temperature (13.4 °C) during seed germination.

The daily mean temperature was 16.36 °C; the mean day-time temperature was 19.57 °C, while the mean night-time temperature was 13.39 °C (Fig. 1).

**Figure 2:**
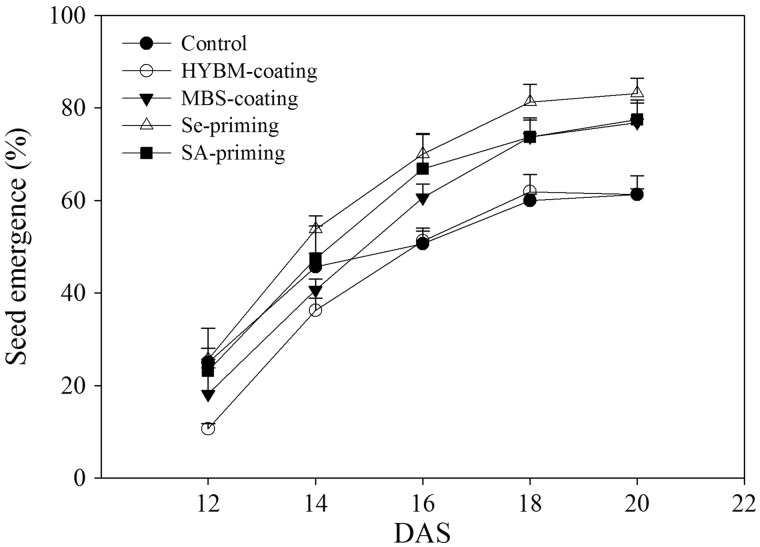
Emergence dynamics of HYBM-coated, MBS-coated, Se-primed, SA-primed and non-treated seeds of direct-seeded early rice in field experiment. (DAS, days after sowing; HYBM coating, Hanyubaomu coating; MBS coating, Miaoboshi coating; Se priming, priming with 50 µM sodium selenite; SA-priming, priming with 100 mg L^−1^ salicylic acid.) The seed emergence data were recorded from 12 DAS till constant at 20 DAS. Error bars indicate standard error (*n* = 4).

In the field conditions, Se priming, SA priming and MBS coating enhanced the seedling emergence, while HYBM coating did not promote the emergence of rice seedlings compared with the non-treated control. In the non-treated control (Fig. 2), the seedling emergence at 12 DAS was 25.00 %, and the final emergence at 20 DAS was only 61.25 %. Compared with the non-treated control, Se priming, SA priming and MBS coating significantly increased the final emergence of the rice seedlings by 26.31 %, 20.96 % and 20.02 %, respectively. The final emergence of HYBM coating was 61.25 %, which was statistically insignificant as compared with control (*P* > 0.05).

In field conditions, the pre-sowing seed treatments enhanced the growth of rice seedlings compared with the non-treated control (Table 1). At 12 DAS, the root length, shoot length, root fresh weight and shoot fresh weight of the non-treated rice seedlings were only 3.93 cm, 4.70 cm, 10.22 mg per seedling and 24.56 mg per seedling, respectively. On an average, the two seed priming treatments significantly increased the root length, shoot length, root fresh weight and shoot fresh weight by 15.73 %, 13.34 %, 20.92 % and 22.52 %, respectively, compared with the non-treated control. The two seed coating treatments also increased the root length, root fresh weight and shoot fresh weight by 7.30 %, 15.50 % and 13.95 %, respectively (except that the root fresh weight of the coated seedlings did not increase). However, most of the improvements were limited and did not achieve significance (*P* ≤ 0.05) (except for the root length in MBS and shoot fresh weight in both seed coating treatments). When comparing the two seed priming treatments, no significant difference (*P* ≤ 0.05) was observed except that the shoot fresh weight for Se priming was greater than for SA priming. Similarly, the effects of the two seed-coating treatments were almost equivalent.

**Table1.** The effects of different pre-sowing seed treatments on seedling growth attributes of direct-seeded early rice at 12 DAS in a field experiment. Different lowercase letters denote statistical differences between treatments of a cultivar at the 5 % level according to LSD test.FW, fresh weight; HYBM, Hanyubaomu; MBS, Miaoboshi; Se, 50 µM sodium selenite; SA, 100 mg L^−1^ salicylic acid.

Pre-sowing treatment	Root length (cm)	Shoot length (cm)	Root FW (mg per seedling)	Shoot FW (mg per seedling)
Control	3.93±0.08 b	4.70±0.15 c	10.22±0.86 b	24.56±0.81 c
HYBM-coating	3.98±0.13 b	4.46±0.14 c	11.84±0.36 ab	27.84±0.99 b
MBS-coating	4.50±0.05 a	4.87±0.16 bc	12.34±0.72 ab	29.05±1.13 b
Se-priming	4.58±0.06 a	5.26±0.25 ab	12.68±0.77 a	34.31±1.41 a
SA-priming	4.75±0.14 a	5.59±0.34 a	13.16±1.34 a	29.25±0.95 b
d*f*	4, 15	4, 15	4, 15	4, 15
*F*	12.98	4.19	1.69	10.54
*P*	<0.01	0.017	0.205	<0.01

### Growth chamber experiment

**Figure 3:**
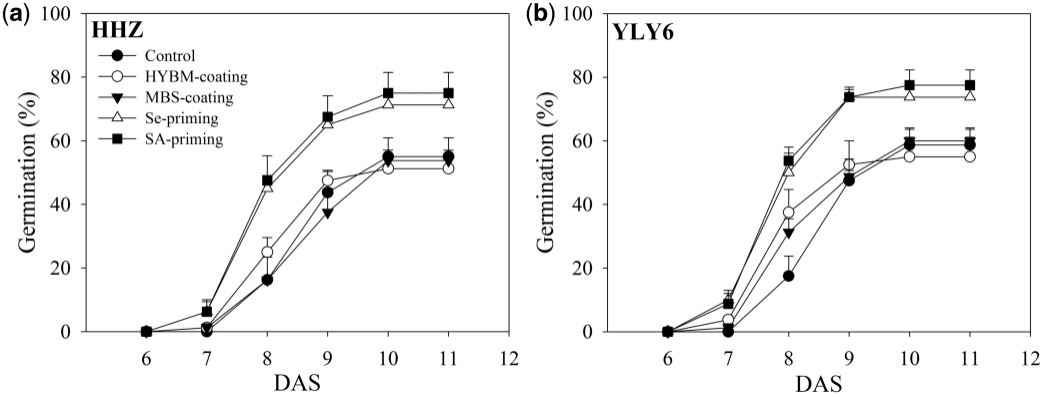
Germination dynamics of HYBM-coated, MBS-coated, Se-primed, SA-primed and non-treated seeds of rice in growth chamber experiment. (a) HHZ. (b) YLY6. (HHZ, Huanghuazhan; YLY6, Yangliangyou6; Control, low-temperature control using non-treated seeds; DAS, days after sowing; HYBM coating, Hanyubaomu coating; MBS-coating, Miaoboshi coating; Se priming, priming with 50 µM sodium selenite; SA priming, priming with 100 mg L^−1^ salicylic acid). The seed germination data were recorded from 3 DAS till constant at 11 DAS. Error bars indicate standard error (*n* = 4).

Based on the temperatures recorded in the field from 1 DAS to 10 DAS, the temperature for the growth chamber experiment was set as 19.6 °C during the day and 13.4 °C during the night. Significant differences were observed in final germination percentage among different pre-sowing seed treatments (*F*_4,24 _=_ _8.90, *P* < 0.01). Se priming and SA priming significantly increased germination (*P* < 0.05), while neither HYBM coating nor MBS coating increased the final germination compared with the control in both cultivars (Fig. 3). The final germination of the control treatment was 55.00 % and 58.75 % in HHZ and YLY6, respectively, When averaged across cultivars, Se priming and SA priming significantly increased the final germination by 21.55 % and 25.41 %, respectively, compared with the control. There was no significant difference in final germination between the control and the seed coating treatments in both cultivars, as the germination rates with HYBM coating and MBS coating were 51.25 % and 53.75 % in HHZ and 55.00 % and 60.00 % in YLY6, respectively. No significant difference was found between the two cultivars In their responses to the pre-sowing seed treatments (*F*_4,24 _=_ _0.05 *P* = 0.99).

**Figure 4:**
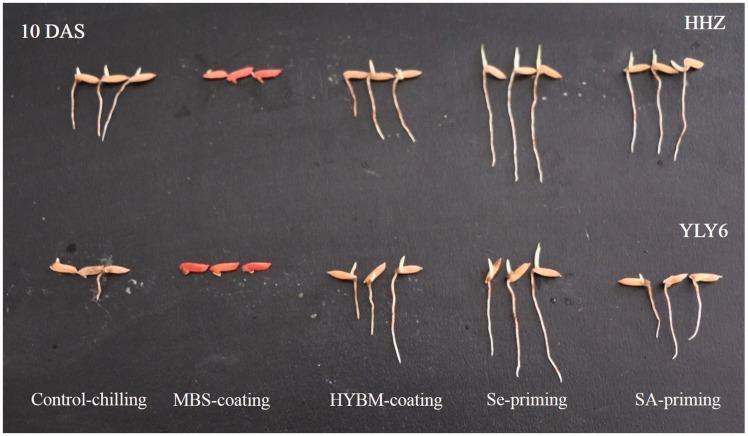
Pictorial illustration of HYBM-coated, MBS-coated, Se-primed, SA-primed and non-treated rice seedlings of HHZ and YLY6 at 10 DAS under chilling stress in a growth chamber experiment. (HHZ, Huanghuazhan; YLY6, Yangliangyou6; Control, low-temperature control using non-treated seeds; DAS, days after sowing; HYBM coating, Hanyubaomu coating; MBS coating, Miaoboshi coating; Se priming, priming with 50 µM sodium selenite; SA priming, priming with 100 mg L^−1^ salicylic acid).

In the chilling stress conditions of the growth chamber experiment, pre-sowing seed treatments enhanced the root length, shoot length, root fresh weight and shoot fresh weight of the rice seedlings in both cultivars as compared with control (Fig. 4 and Table 2). Both seed-priming treatments significantly enhanced the average seedling growth of both HHZ and YLY6. Se priming and SA priming increased the root length, shoot length, root fresh weight and shoot fresh weight of the rice seedlings by averages of 25.77 %, 46.11 %, 28.25 % and 33.52 %, respectively, compared with the control. The two seed coating treatments also enhanced the seedling growth, but their effects were far inferior to the effects of the seed priming treatments. When compared with the control, seed coating increased the root length, shoot length, root fresh weight and shoot fresh weight of the rice seedlings by 12.37 %, 15.38 %, 15.80 %, 12.06 %, respectively. However; most of the improvements induced by the seed coating treatments were statistically insignificant (*P* ≤ 0.05) as compared with non-treated control. Based on the *F* ratios of two-way ANOVAs (Table 2), the variances in their response to pre-sowing seed treatments were insignificant between the two rice varieties.

**Table 2.** Seedling growth attributes of two rice cultivars under the influence of pre-sowing seed treatments and chilling stress at 12 DAS in growth chamber experiment. *Different lowercase letters denote significant differences between treatments of a cultivar at the 5 % level according to LSD test.DAS, days after sowing; Control, low-temperature control using non-treated seeds; HYBM coating, Hanyubaomu coating; MBS coating, Miaoboshi coating. The ratio of seed weight to coating agent weight was 8:1 for HYBM and 40:1 for MBS; Se priming, priming with 50 µM sodium selenite; SA priming, priming with 100 mg L^−1^ salicylic acid.

Variety	Pre-sowing treatment	Root length (cm)	Shoot length (cm)	Root FW (mg per seedling)	Shoot FW (mg per seedling)
Huanghuazhan	Control	3.73±0.19 c	1.48±0.16 c	10.33±0.31 c	11.57±0.47 b
	HYBM-coating	4.27±0.20 b	1.94±0.13 b	13.83±0.89 ab	14.77±0.75 b
	MBS-coating	4.05±0.20 bc	1.76±0.13 bc	11.83±0.79 bc	13.94±2.33 b
	Se-priming	5.16±0.21 a	2.86±0.09 a	15.71±0.97 a	19.76±1.38 a
	SA-priming	5.12±0.18 a	2.92±0.11 a	15.61±1.15 a	19.15±1.23 a
Yangliangyou-6	Control	4.42±0.39 c	2.16±0.17 b	15.30±0.16 c	18.52±1.23 c
	HYBM-coating	5.11±0.89 b	2.44±0.19 b	17.70±1.02 b	21.17±1.09 bc
	MBS-coating	5.17±0.25 b	2.45±0.29 b	17.54±0.68 b	18.54±1.34 bc
	Se-priming	5.61±0.29 ab	3.72±0.45 a	19.03±0.52 ab	24.08±2.11 ab
	SA-priming	6.05±0.27 a	3.92±0.12 a	20.46±1.07 a	26.31±1.49 a
Treatments	d*f*	4, 24	4, 24	4, 24	4, 24
	*F*	19.84	31.41	21.38	14.07
	*P*	<0.01	<0.01	<0.01	<0.01
Varieties	d*f*	1, 3	1, 3	1, 3	1, 3
	*F*	13.80	10.24	15.54	25.43
	*P*	0.03	0.05	0.03	0.02
Treatments* varieties	d*f*	4, 24	4, 24	4, 24	4, 24
	*F*	0.82	0.55	4.09	0.54
	*P*	0.52	0.70	0.38	0.71

**Figure 5:**
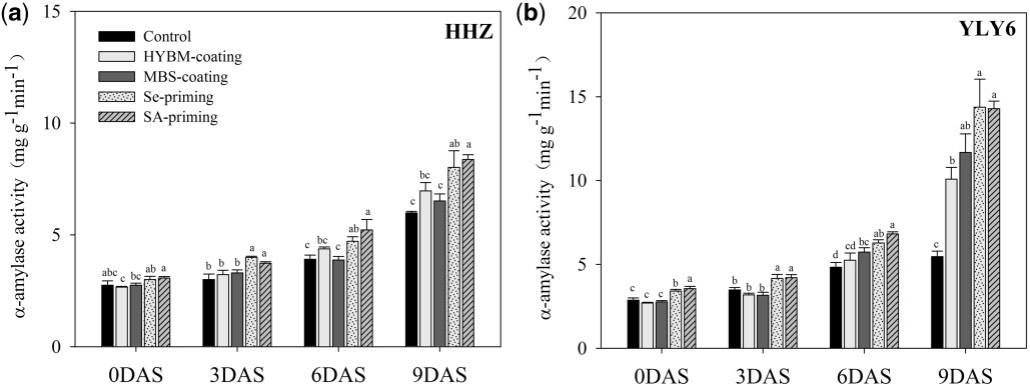
Variations in α-amylase activity of rice seeds and seedlings in HYBM-coated, MBS-coated, Se-primed, SA-primed and non-treated seed treatments at 0, 3, 6 and 9 DAS in growth chamber experiment. (a) HHZ. (b) YLY6. (HHZ, Huanghuazhan; YLY6, Yangliangyou6; Control, low-temperature control using non-treated seeds; DAS, days after sowing; HYBM coating, Hanyubaomu coating; MBS coating, Miaoboshi coating; Se priming, priming with 50 µM sodium selenite; SA priming, priming with 100 mg L^−1^ salicylic acid). Seeds at 0 DAS were tested on a dry weight basis, while seedlings at 3, 6 and 9 DAS were tested on a fresh weight basis. Different lowercase letters denote significant differences among treatments of a cultivar at the 5 % level according to LSD test. Error bars above mean indicate standard error (*n* = 4).

**Figure 6:**
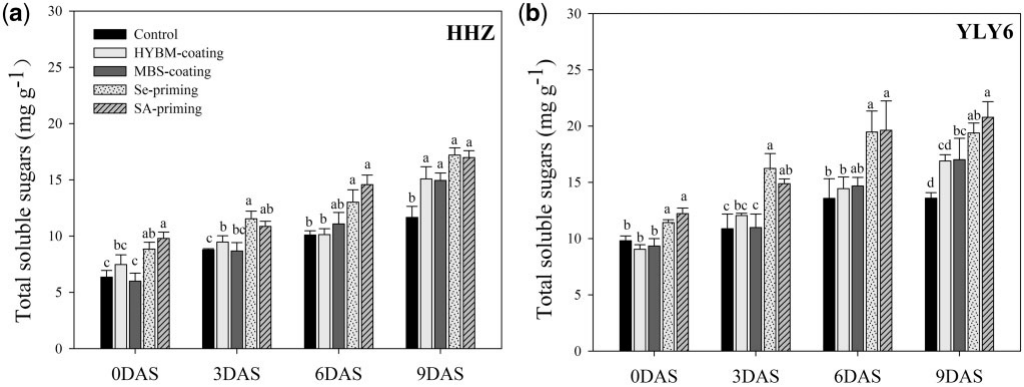
Variations in total soluble sugar contents of rice seeds and seedlings in HYBM-coated, MBS-coated, Se-primed, SA-primed and non-treated seed treatments at 0, 3, 6 and 9 DAS in growth chamber experiment. (a) HHZ. (b) YLY6. (HHZ, Huanghuazhan; YLY6, Yangliangyou-6; Control, low-temperature control using non-treated seeds; DAS, days after sowing; HYBM coating, Hanyubaomu coating; MBS coating, Miaoboshi coating; Se priming, priming with 50 µM sodium selenite; SA priming, priming with 100 mg L^−1^ salicylic acid). Seeds at 0 DAS were tested on a dry weight basis, while seedlings at 3, 6 and 9 DAS were tested on a fresh weight basis. Different lowercase letters denote significant differences among treatments of a cultivar at the 5 % level according to LSD test. Error bars above mean indicate standard error (*n* = 4).

The starch metabolism of the rice dry seeds and seedlings was assessed in terms of α-amylase activity and total soluble sugars. Significant differences were existed among different pre-sowing treated seeds and seedlings on α-amylase activity and total soluble sugars at 0DAS (α-amylase activity: *F*_4,24 _=_ _17.39 *P* < 0.01; total soluble sugars: *F*_4,24 _=_ _10.88 *P* < 0.01), 3DAS (α-amylase activity: *F*_4,24 _=_ _24.39 *P* < 0.01; total soluble sugars: *F*_4,24 _=_ _9.01 *P* < 0.01), 6DAS (α-amylase activity: *F*_4,24 _=_ _15.74 *P* < 0.01; total soluble sugars: *F*_4,24 _=_ _7.83 *P* < 0.01) and 9DAS (α-amylase activity: *F*_4,24 _=_ _21.68 *P* < 0.01; total soluble sugars: *F*_4,24 _=_ _11.71 *P* < 0.01) (Figs 5 and 6). The data revealed that both of these attributes progressively increased and achieved their highest values at 9 DAS. In both cultivars, seed priming treatments significantly increased the α-amylase activity and total soluble sugar contents of the dry seeds (0 DAS) and the rice seedlings (3, 6, 9 DAS) compared with the control, while for the seed coating treatments, the enhancements of α-amylase activity and total soluble sugar contents were not significant in most cases. Averaging over the priming treatments and the cultivars, the α-amylase activity and total soluble sugar contents of the primed dry seeds (0 DAS) increased by 13.57 % and 24.39 %, respectively, as compared with the control, and the starch metabolism advantages of seed priming were maintained from 3 DAS to 9 DAS. At 9 DAS, the Se priming treatment increased the α-amylase activity and total soluble sugar contents by 43.71 % and 31.04 %, while the SA priming treatment produced increases of 45.17 % and 32.97 %, respectively, compared with the control. However, the same results were not observed for the seed coating treatments. In both cultivars, neither HYBM coating nor MBS coating increased the α-amylase activity and total soluble sugar contents of the dry seeds (0 DAS) or the 3 DAS or 6 DAS rice seedlings (except that MBS coating significantly increased the α-amylase activity of 6 DAS rice seedlings in YLY6), while at 9 DAS, the starch metabolism of the coated seedlings was progressively enhanced compared with the control (the change in α-amylase activity in HHZ and total soluble sugar contents in YLY6 did not achieve significance (*P* ≤ 0.05). When averaged across the seed coating treatments and the cultivars, seed coating increased the α-amylase activity and the total soluble sugar contents of the 9 DAS rice seedlings by 30.52 % and 21.11 %, respectively, compared with the control. The changes of α-amylase activity and total soluble sugar contents in the rice seeds and seedlings induced by pre-sowing seed treatments were similar between HHZ and YLY6, suggesting no interaction effects between varieties and seed treatments.

## Discussion

In the present study, seed priming was effective in enhancing chilling tolerance, where better germination and early seedling growth was observed in both Se priming and SA priming as compared with non-treated control (Figs 2 and 3; Tables 1 and 2). Previous studies have reported seed priming was effective in inducing low-temperature tolerance during seed germination (He *et al.* 2002; Sasaki *et al.* 2005). He *et al.* (2002) reported significantly higher and faster germination of primed rice seeds under low-temperature (5 °C) stress. Sasaki *et al.* (2005) noted that seedling growth under low temperature in a greenhouse was promoted by priming treatments. Most of the previous studies were conducted under wet-direct-seeded conditions or only in tray experiments, whereas our studies implied that in dry direct-seeded rice, the effects of priming treatments on seed germination and seedling growth were also pronounced and stable under chilling stress. In this study, the selection of Se priming and SA priming treatments was based on a series of preliminary studies in which we found that Se and SA priming treatments were more effective for enhancing chilling tolerance than hydropriming (primed with distilled water). These results reflected that the priming agents salicylic acid and sodium selenite could enhance chilling resistance at low temperature.

Previous studies have reported the positive roles of SA- and Se-priming on increasing stress resistance. Pouramir *et al.* (2014) examined the effect of SA priming on rice seedling growth under chilling stress at seedling stage, and suggested that SA priming could improve the chilling tolerance of rice seedlings by increasing the activity of antioxidant enzyme. However, the SA priming-induced chilling tolerance during rice seed germination was not studied in Pouramir’s work. Khaliq *et al.* (2015) reported the effect of Se priming on seed germination and seedling growth, however, the experiments were conducted under normal conditions (without any stress). From Khaliq’s study, we may not deduce the effect of Se priming on rice seed germination and associated physio-biochemistry changes under chilling stress. While in our study, the main focus was to examine the effect of seed treatments on rice seed germination and the associated mechanisms during seed germination stage under chilling stress.

The two seed coating treatments slightly enhanced the growth of rice seedlings under chilling stress, but their effects were limited. Under field conditions, MBS coating increased the final emergence of the rice seedlings, while HYBM coating did not (Fig. 2 and Table 1), however, in the growth chamber experiment, neither HYBM coating nor MBS coating enhanced seed germination in either cultivar (Fig. 3). Similar observations have also been reported in previous studies. Xiao (2010) reported that MBS increased the seedling emergence rate by 6–16 %. Zeng and Shi (2009) reported that seed coating treatment could stimulate the emergence of rice seedlings and increase their root activity, while other research showed that seed coating treatment could negatively affect seed germination to some extent (Scott, 1987; Mori *et al.* 2012). Generally, coating agents consist of polymer materials and chemical components such as pesticides and plant growth regulators (Kaufman, 1991). The impermeability of the polymer materials will influence the gas and water exchange from the soil to the seeds, and the loss of coating agents from seeds after rain could dilute or delay the effects of coating agents on seed germination and seedling growth. The reasons for the differences between the field and growth chamber experiments on seed germination still require further study.

In this study, seed priming treatments significantly enhanced the starch metabolism, while the improvement induced by seed coating treatments was limited (Figs 4 and 5). higher α-amylase activity and total soluble sugar contents in primed seeds and seedlings were closely related to the higher seed germination/emergence and faster seedling growth compared with the control, possibly because starch degradation was activated and seed reserves mobilization was greatly improved by seed priming treatments under chilling stress. The ability of plants to degrade starch into soluble sugars most likely plays a key role in their ability to survive and grow faster under a wide range of environments. In rice, amylase activity is greatly induced during germination (Tanaka *et al.* 1970). The high α-amylase activity in primed seeds and seedlings is also reflected through higher soluble sugar concentrations and a faster rate of starch breakdown in germinating primed seeds, which presumably provided the substrates necessary to generate the energy required for growth and maintenance processes. It has also been proposed that the degradation and conversion of seed reserves during the germination process are related to the increase in soluble sugar contents, which presumably provides the substrates necessary to generate the energy required for growth and maintenance processes (Hussain *et al.* 2015). In addition to the mechanisms associated with starch metabolisms, the chilling tolerance during rice seed germination and early seedling growth induced by seed treatments might be attributed to increased level of antioxidant activity and greater membrane stability (Pouramir *et al.* 2014; Khaliq *et al.* 2015). Pouramir *et al.* (2014) reported that the positive role of SA priming in chilling resistance in rice seedlings was associated with increased level of antioxidant activity in primed seeds. Khaliq *et al.* (2015) found that Se priming improved crop establishment because of greater membrane stability and increased activity of antioxidants induced by Se priming besides improved starch metabolism. Although seed coating did not enhance starch metabolism from 0 DAS to 6 DAS, a noticeable increase in α-amylase activity and total soluble sugar contents was observed at 9 DAS compared to the control, which indicated that seed coating might have a positive influence on seedling growth during the seedling stage. Besides, exploring the α-amylase genes or carbohydrate remobilization genes induced by seed treatment may deeply disclose the mechanism of these seed treatments. We are keenly focusing to carry out the deep transcriptomic/proteomic studies in near future.

## Conclusions

Our study conclusively revealed that Se priming and SA priming treatments significantly enhanced the seed germination and seedling growth of dry direct-seeded early rice under chilling stress. The improved emergence and vigorous seedling growth induced by the seed priming treatments were associated with increased α-amylase activity and total soluble sugar contents in the primed seedlings. These findings will have practical implications for the sustainability and productivity of dry direct-seeded early rice. 

## Sources of Funding

This work is supported by the National Natural Science Foundation of China (Project No. 31371571), the National High Technology Research and Development Program of China (863 Program) (Project No. 2014AA10A605) and Special Fund for Agro-scientific Research in the Public Interest of China (Project No. 201203096).

## Contributions by the Authors

W.W. and L.N. initiated and designed the research, W.W., J.M., H.D. and Q.C. performed the experiments, W.W. and L.N. analysed the data and wrote the article, S.P. revised and edited the article and also provided advice on the experiments. Each author has seen and agreed to the submitted article.

## Conflict of Interest Statement

None declared.
